# Supplementary figures and images for: Immunological Profiles in Parry–Romberg Syndrome: A Case–Control Study
Source: J Clin Med. 2024 Feb 21;13(5):1219. doi: 10.3390/jcm13051219 (PMC10932088; doi:10.3390/jcm13051219)

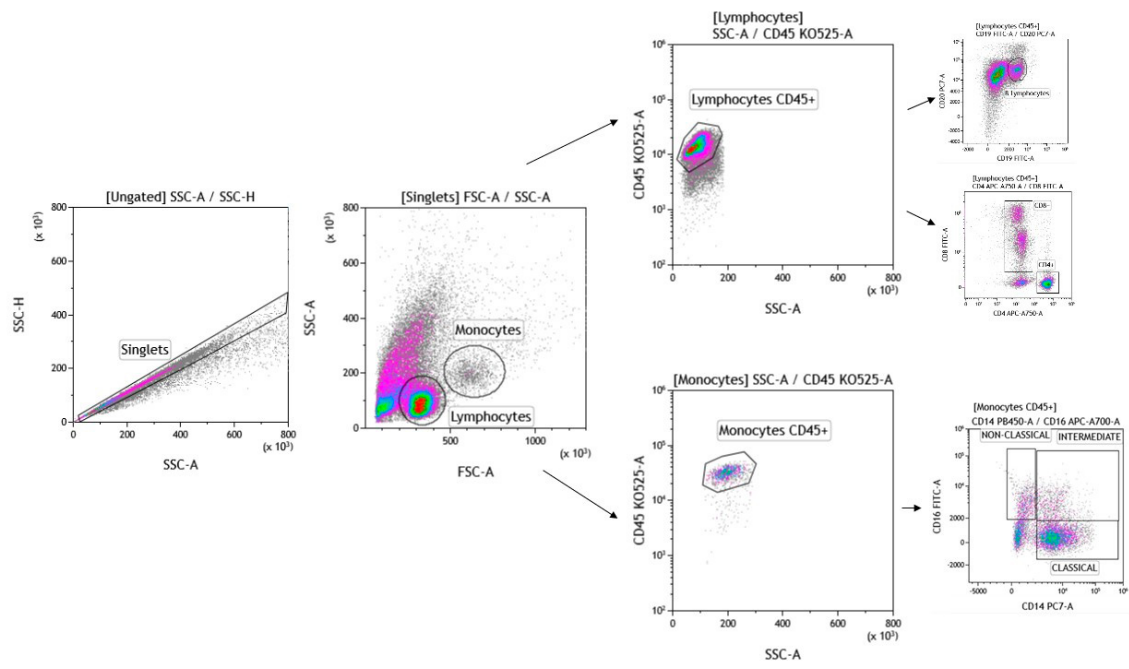

**Figure S1.** Gating strategies.

Supplement: Supplementary file 1 [file jcm-13-01219-s001.zip › jcm-2846165-supplementary.pdf]
